# Beyond Comorbidity: Pulmonary Adenocarcinoma in a Patient with Rheumatoid Arthritis—A Case Report and Literature Review

**DOI:** 10.3390/life15071118

**Published:** 2025-07-17

**Authors:** Ancuța-Alina Constantin, Mihai Alexandru Arghir, Dana Avasilcăi, Florin-Dumitru Mihălțan

**Affiliations:** 1 Department of Cardio-Thoracic Pathology, “Carol Davila” University of Medicine and Pharmacy, 050474 Bucharest, Romania; florin.mihaltan@umfcd.ro; 2 Institute of Pneumology “Marius Nasta”, 050159 Bucharest, Romania; marghir117@gmail.com (M.A.A.);

**Keywords:** lung cancer, rheumatoid arthritis, risk factors, malignancy, methotrexate

## Abstract

Lung cancer is one of the most common and deadly forms of cancer worldwide, despite sustained efforts to encourage smoking cessation and raise awareness of the risk factors. In Romania, lung cancer is a significant health challenge, being the leading cause of death caused by cancer, especially amongst men. The incidence of lung cancer in connective tissue disease (CTD) varies in different studies from 4.5% in rheumatoid arthritis (RA), to 4.4% in polymyositis or dermatomyositis, and up to 11.1% in systemic sclerosis. However, older studies have shown an increased risk of cancer in patients with rheumatoid arthritis (RA), ranging from 10% to 30% compared to the general population, particularly in those undergoing methotrexate therapy. Rheumatoid arthritis affects approximately 40 per 100,000 people annually worldwide, with a three- to four-fold higher incidence in women. Non-small cell lung cancer (NSCLC), the most common lung cancer subtype, has been linked to RA, yet the association remains poorly defined, with limited insight into the underlying molecular mechanisms. We present the case of a 61-year-old male with a 49-pack-year smoking history and a known diagnosis of rheumatoid arthritis, currently managed with methotrexate therapy. He was admitted for evaluation due to a progressive decline in general condition, characterized by worsening dyspnea and chest pain, symptoms that had been longstanding but had markedly exacerbated over the past two weeks. Based on a chest CT performed prior to the patient’s admission to our clinic, subsequent diagnostic investigations established the diagnosis of pulmonary adenocarcinoma. The diagnostic process proved to be particularly challenging due to the presence of multiple comorbidities, which significantly impacted both the diagnostic approach and the overall clinical trajectory.

## 1. Introduction

Lung adenocarcinoma forms in the glandular or secretory cells localized in alveoli and bronchioles and is the most common histological subtype of NSCLC, accounting for approximately 40–50% of all lung cancers cases [1]. It typically arises in the peripheral regions of a lung and it is the most common subtype that affects non-smokers or light smokers, especially females [2]. This form of NSCLC is characterized by considerable heterogeneity across the histological, cellular, and molecular dimensions, which contributes to diverse clinical presentations, progression patterns, and responses to therapy [3]. Rheumatoid arthritis is the most common systemic autoimmune disease [4], primarily targeting synovial joints, but often presenting extra-articular manifestations due to widespread immune dysregulation. Emerging evidence suggests that prolonged immune dysregulation and the sustained inflammatory response characteristic of RA may play a central role in increasing the risk of cancer development [5]. Among the severe complications associated with RA is an increased risk of malignancy, particularly lung cancer and lymphoma [6]. Various risk determinants for the development of lung cancer in the context of RA have been proposed, including male sex, smoking, and the presence of interstitial lung disease or some therapies, including Disease-Modifying Antirheumatic Drugs (DMARDs) [7,8]. Smoking represents a common risk factor for both conditions, accounting for 80–90% of lung cancer cases [9] and also conferring a 40% increased risk of developing rheumatoid arthritis among individuals who have ever smoked, compared to non-smokers [10]. However, the underlying mechanisms linking RA to increased lung cancer risk remain insufficiently understood [5,7].

A multidisciplinary approach and a multidimensional strategy are essential for an accurate diagnosis in complex presentations, which involves combining thorough anamnesis, detailed imaging studies, and advanced diagnostic procedures like tissue biopsies. Attention should be especially directed towards the differential diagnosis between pleural metastases and mesothelioma, which may have similar radiological features with these other types but differ significantly in prognosis, treatment, and underlying pathology. Additionally, the existence of multiple comorbidities (especially those related to autoimmune diseases such as rheumatoid arthritis) adds another layer of complexity. Besides affecting clinical presentation and radiologic interpretation, these comorbid conditions may also alter disease progression through unexplored mechanisms.

## 2. Detailed Case Description

We report the case of a 61-year-old male, with a smoking index of 49-pack-years and prolonged occupational exposure to respiratory irritants due to 10 years of work in construction, who presented to our medical department with complaints of mixed-pattern dyspnea with minimal exertion, and a non-radiating quasi-permanent thoracic pain localized to the middle posterior third of the right hemithorax, of moderate to high intensity. Additional symptoms included a seromucous cough, a subjective fever not documented by measurement at home, decreased appetite, and an unintentional weight loss of approximately 4 kg over the preceding month. Medical history reveals that the patient has a long-standing diagnosis of chronic obstructive pulmonary disease (COPD), currently treated with an inhaled dual bronchodilator therapy consisting of a long-acting muscarinic antagonist (LAMA) and a long-acting beta-2 agonist (LABA).

**Figure 1:**
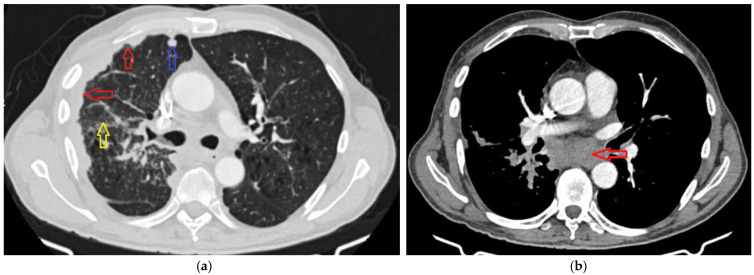
Right pleural thickening with an irregular, pseudonodular appearance and a small right apical pulmonary nodule (**a**); infracarinal adenopathic mass measuring 48 × 26 mm (**b**).

In addition, the patient has seropositive RA, managed with methotrexate, and valvular heart disease characterized by mild mitral and tricuspid regurgitation. In the context of the known rheumatologic disease, a contrast-enhanced chest CT scan was performed in February 2025, revealing a polymorphic lesion pattern, including a pseudonodular thickening of the right pleuro-parietal region (Figure 1a—red arrow), suggesting the possibility of mesothelioma, as well as other nodular pulmonary lesions, more prominently visible in the upper lobe of the right lung (Figure 1a—blue arrow). Additionally, discrete interstitial changes through interstitial thickening (Figure 1a—yellow arrow) and carinal adenopathy were identified, measuring 48 × 26 mm (Figure 1b—red arrow), along with panlobular emphysematous changes and perihilar cylindrical bronchiectasis. Previous investigations conducted in local healthcare facilities failed to determine the underlying etiology of these radiological findings.

During the physical examination, the patient was hypoxemic, with an oxygen saturation of 89% in room air. Pulmonary auscultation showed decreased vesicular breath sounds in the middle third of the right hemithorax, without any additional adventitious sounds. The patient was afebrile at presentation and had a body mass index (BMI) of 19 kg/m^2^.

Upon admission, extensive investigations were performed to assess the patient’s hemodynamic and respiratory statuses. An arterial blood gas analysis (Table 1) revealed hypoxemic, normocapnic, type 1 respiratory failure. In this case, immediate administration of oxygen therapy at a flow rate of 2.5 L per minute was initiated, resulting in a slow improvement of dyspnea. The laboratory tests (Table 2) showed leukocytosis with neutrophilia (WBC = 16.55 × 10^3^/µL, NEUT = 13.70 × 10^3^/µL); biological inflammatory syndrome (ESR = 33 mm/h; fibrinogen = 555 mg/dL; CRP = 138 mg/L); an elevated LDH level (809 U/L); and minimal iron deficiency (Serum Iron = 32.8 ng/dL). Therefore, empirical antibiotic therapy was administered with ceftriaxone at dosage of 1 g × 2/day. A urine culture subsequently identified Enterococcus faecalis, prompting an optimization of the antibiotic regimen through the addition of linezolid, based on the antibiogram results.

Pulmonary functional testing, performed through spirometry, revealed a very severe mixed respiratory dysfunction (Table 3), which can be explained in this case from the association of COPD, which causes the obstructive component and pleural thickening that leads to a limitation of lung expansion and reduced compliance, with a significant impact on vital capacity.

**Figure 2:**
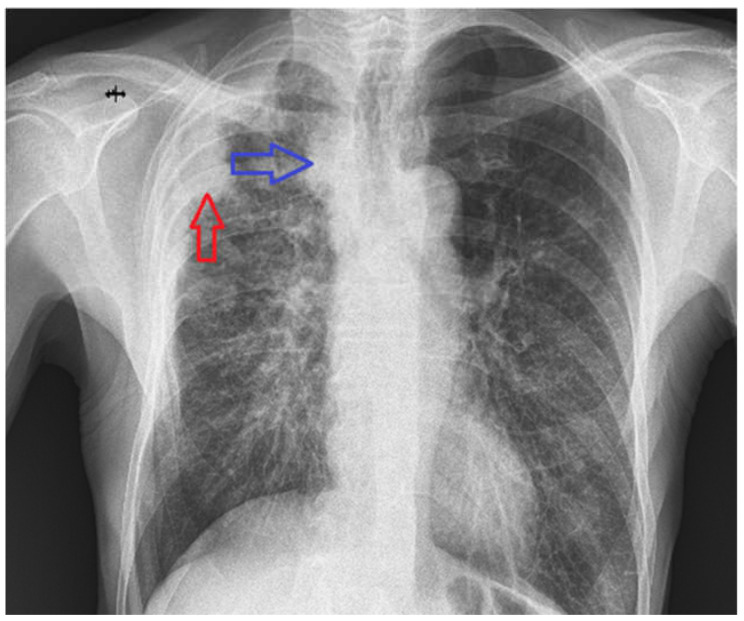
PA chest X-ray—homogeneous opacity, adherent to the right lateral thoracic wall in the upper third and an increased right mediastinal shadow.

The primary radiologic investigation performed in our clinic was a posteroanterior chest X-ray (Figure 2), which revealed a homogeneous opacity adherent to the right lateral thoracic wall in the upper third (Figure 2—red arrow), a nonspecific widening of the right mediastinum (Figure 2—blue arrow), and also a discreet accentuation of the peribronchovascular markings in the right infrahilar region.

Given these preliminary imaging findings, along with our patient’s nonspecific thoracic pain—previously attributed to rheumatoid arthritis—and the persistence of dyspnea and hypoxemia, which remained refractory to oxygen therapy, with only partial improvement in the arterial blood gas parameters, a contrast-enhanced chest CT scan was warranted and subsequently performed.

**Figure 3:**
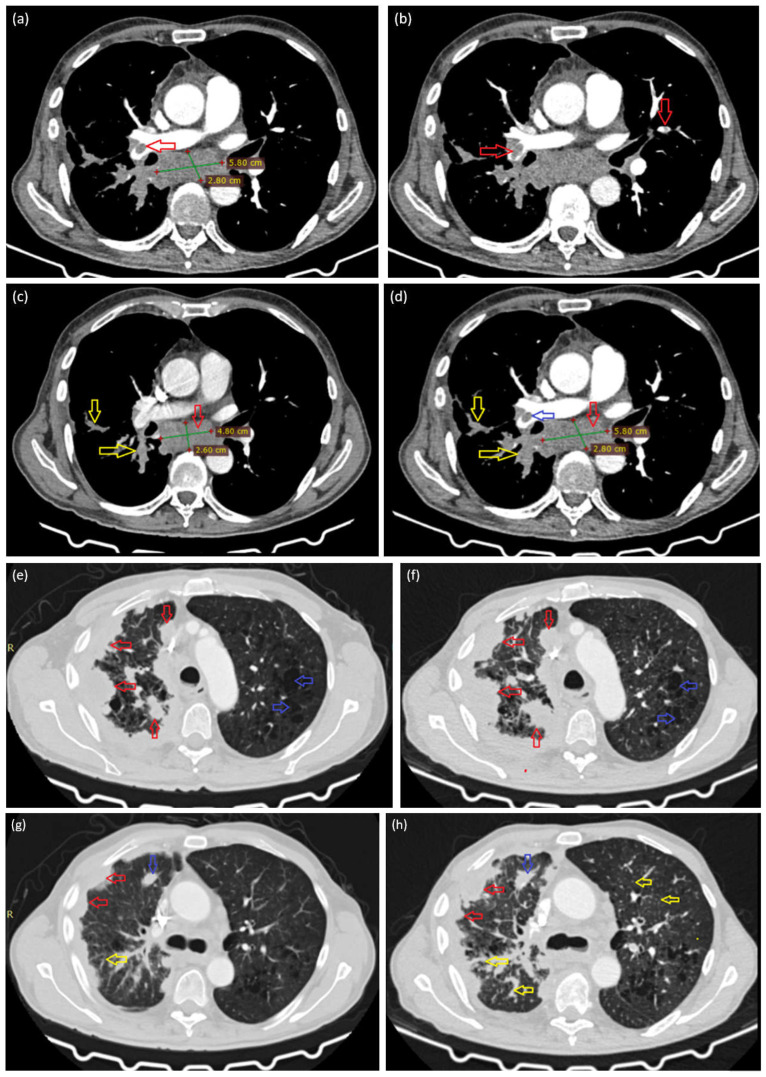
Central embolus in the right pulmonary artery, and infracarinal lymph node (**a**). Bilateral pulmonary arterial filling defects, both central and peripheral and subocclusive and occlusive in nature (**b**). Comparative imaging assessment between the lesions documented one month prior to admission ((**c**,**e**,**g**)—left-sided images) and those identified during the current evaluation at our clinic ((**d**,**f**,**h**)—right-sided images).

The current CT examination, correlated with the previous investigation from another medical facility, reveals significant bilateral pulmonary arterial filling defects, both subocclusive and occlusive, predominantly on the right side (Figure 3a—red arrows; Figure 3b—red arrows, Figure 3d—blue arrows). In addition to the bilateral pulmonary thromboembolism, the investigation also indicates a progression of the lesions previously identified on the CT scan performed one month earlier, as evidenced by an increase in the size of the infracarinal lymphadenopathy from 48 × 26 mm to approximately 58 × 28 mm (Figure 3c,d—red arrows). Persistent iodophilic polynodular and lamellar pleural thickenings are also noted on the right side, extending along the peribronchovascular structures from the hilar region (Figure 3c,d—yellow arrows, Figure 3e,f—red arrows). Furthermore, the lung parenchyma demonstrates an emphysematous appearance (Figure 3e,f—blue arrows), with the presence of pulmonary micronodules and interstitial thickening (Figure 3g,h—yellow arrows).

Given the confirmed diagnosis of a pulmonary embolism, a cardiology consultation was warranted. Due to the clinical and imaging findings, a switch from prophylactic to therapeutic anticoagulation was considered necessary, as the patient had initially been on prophylactic treatment only.

**Figure 4:**
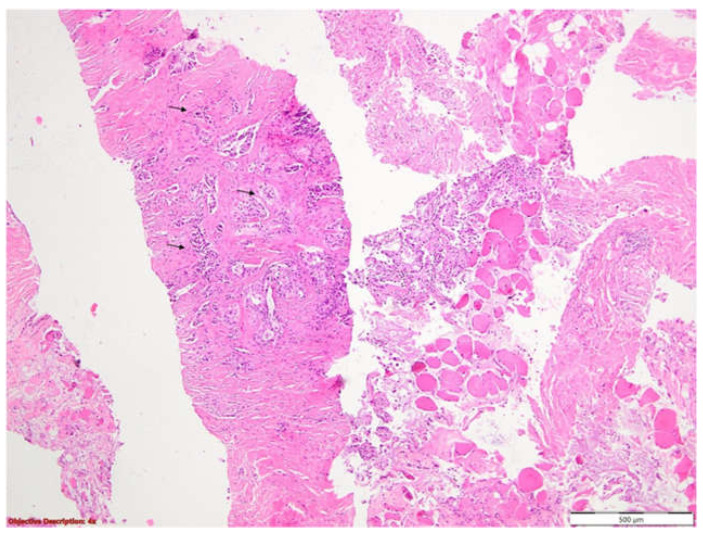
Skeletal muscle fibers and parietal pleura with tumoral proliferation displaying both acinar and solid architectural patterns, suggestive of adenocarcinoma (black arrows); 40×.

**Figure 5:**
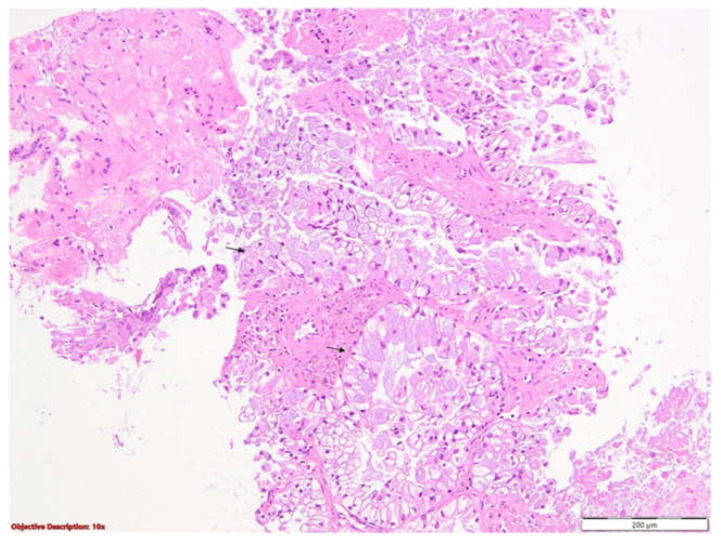
Parietal pleura with adenocarcinoma proliferation composed of large cylindrical cells exhibiting abundant intracytoplasmic mucin and nuclear hyperchromasia (black arrows); HE, 100×.

To clarify the etiology of the pleural abnormalities identified on imaging, a thoracic surgery consultation was obtained, followed by an ultrasound-guided right pleural biopsy. A histopathological examination (Figure 4 and Figure 5) revealed carcinomatous infiltrates with a solid and tubular architecture, composed of atypical cylindrical epithelial cells with mucin secretion. These findings were consistent with a parietal tumor mass, suggestive of adenocarcinoma of an uncertain primary origin, most likely pulmonary. Subsequent immunohistochemical profiling confirmed the diagnosis of invasive pulmonary adenocarcinoma, displaying a predominantly acinar pattern with focal solid components.

**Figure 6:**
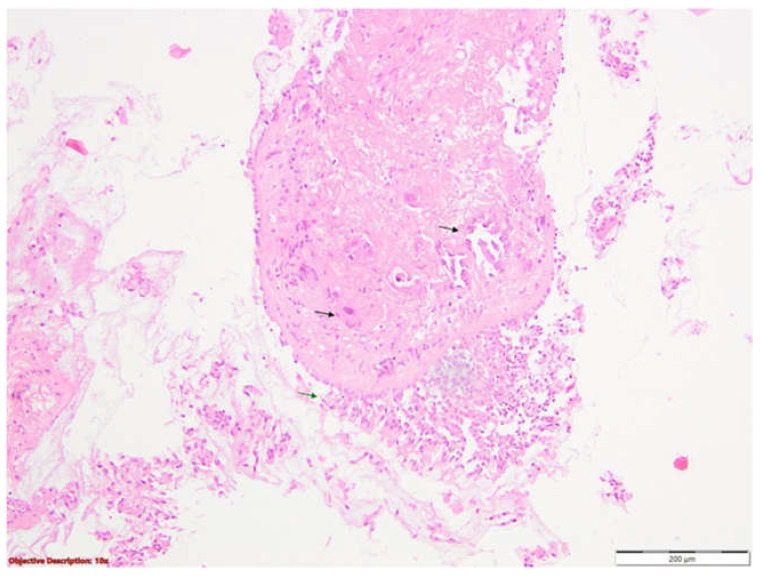
Non-small cell carcinoma, favoring adenocarcinoma. Bronchial mucosa (black arrows →) with tumoral cell infiltration (green arrows →) arranged in groups and acinar-like structures delineated by large neoplastic epithelial cells; HE, 100×.

A bronchoscopy was also performed. An examination of the right bronchial tree revealed a normal configuration of the right main bronchus. The spur of the right upper lobe bronchus appeared slightly widened, while the intersegmental spurs of the right upper lobe (RUL) were prominent, with visibly congested mucosa. The anterior segment of the RUL demonstrated approximately 50% luminal narrowing, which impeded further advancement of the bronchoscope at this level. A biopsy was obtained from the affected area, yielding seven tissue fragments. The histopathological analysis was consistent with poorly differentiated bronchial carcinomatous infiltrates of the non-small cell type, favoring the diagnosis of adenocarcinoma (Figure 6). The next step involved immunohistochemical testing to determine the histogenesis of the tumor. The results subsequently confirmed the diagnosis of invasive pulmonary adenocarcinoma, exhibiting both acinar and solid growth patterns. The tumor showed positive immunoreactivity for TTF1, Napsin A, and CK7, and was negative for p40 and WT1. Additionally, PD-L1 expression was negative in this case.

**Figure 7:**
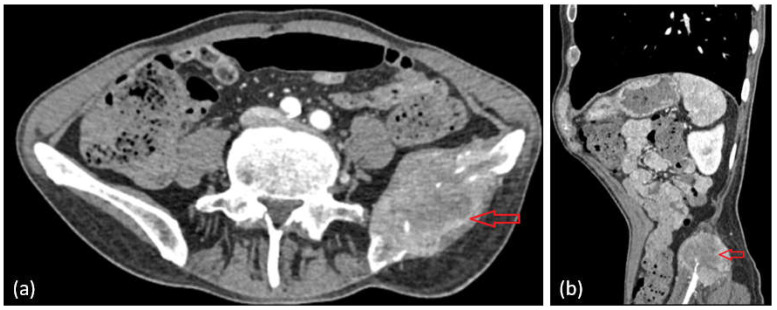
Secondary osseous involvement of the left iliac bone, exhibiting irregular features and extending into the iliacus and gluteus medius muscles (**a**,**b**).

The oncological imaging workup required a contrast-enhanced CT scan of the abdomen and pelvis, which revealed a tumor formation of heterogeneous density, with irregular iodine uptake and poorly defined borders, characterized by invasive lytic behavior involving the left iliac bone and adjacent soft tissues, including the iliac and gluteus medius muscles (Figure 7a,b—red arrow).

Shortly thereafter, the patient’s condition deteriorated, with an onset of severe pain in the right hip, radiating to the posterior side of the left lower limb and with partial relief by the administered analgesic treatment. Given the presence of pleural and pulmonary imaging abnormalities, the tumoral lesion identified in the left iliac region—characterized by its invasive, lytic appearance and the involvement of adjacent soft tissues—is highly suggestive of a secondary (metastatic) process. Nonetheless, histopathological confirmation through biopsy and immunohistochemical analysis is necessary to determine the exact nature of the lesion. We note that, at the moment of this writing, we do not have a biopsy of this iliac lesion.

The diagnosis of pulmonary adenocarcinoma with pleural invasion and secondary bone involvement was established through the integration of clinical and paraclinical data, including the patient’s symptoms, imaging findings, and especially the histopathological and immunohistochemical results. The patient was discharged after diagnosis and clinical stabilization and was referred to specialized oncology services for a comprehensive evaluation and the initiation of targeted therapy.

## 3. Discussion

Lung adenocarcinoma exhibits extensive heterogenicity across the histological, cellular, and molecular domains, contributing to a high variability in clinical presentation, progression, and therapeutic response. One factor which further complicates our patient’s prognosis, besides the disease itself, is his complex medical history, particularly the presence of COPD and rheumatoid arthritis. Not only do these conditions cause a higher risk of lung cancer when compared with unaffected patients, but they also may decrease the efficiency of treatment. Although both COPD and lung cancer are primarily attributed to cigarette smoking, an increasing body of evidence indicates that the association between them extends beyond this shared risk factor. COPD has been identified as an independent risk factor for lung cancer [11], with smokers who exhibit airflow obstruction facing up to a five-fold greater risk of developing lung cancer compared to individuals with normal pulmonary function [12]. The notably high incidence of lung cancer among patients with COPD suggests the presence of overlapping pathogenic mechanisms, potentially involving premature lung aging, genetic predisposition, or shared biological processes mediated by growth factors, intracellular signaling pathways, and epigenetic alterations [13].

In histopathological studies, Schiavon et al. observed that adenocarcinomas associated with COPD exhibited less invasive features, such as a greater lepidic component and lower cellular proliferation, compared to adenocarcinomas in patients without COPD [14]. Conversely, Murakami et al. reported that cancers arising in emphysematous lungs tend to be more aggressive, potentially due to the upregulation of matrix metalloproteinases—enzymes commonly elevated in emphysema—which are linked to lymphovascular invasion and postoperative recurrence [15]. Further supporting this, the post-hoc analyses from two CT screening studies showed that smokers with impaired lung function had pulmonary nodules with shorter volume doubling times (indicating more aggressive behavior) and a lower incidence of indolent lung cancers. This suggests that COPD may serve as a clinical indicator of more aggressive lung cancer [16]. At the molecular level, several studies have found that EGFR mutations and ALK rearrangements are less common in COPD-related lung cancer, with EGFR mutations showing an inverse relationship with the severity of airflow limitation. In contrast, KRAS mutations appear to be unrelated to COPD status. However, it is important to note that these molecular differences may partly reflect the clinical characteristics of the patients involved [17].

In a nationwide cohort study comprising 51.899 patients, Mi Hee Cho et al. showed an increased risk of lung cancer in patients with rheumatoid arthritis. Over a 4.5-year follow-up period, the patients with rheumatoid arthritis had an adjusted hazard ratio of 1.49 (95% CI: 1.34–1.66) for developing lung cancer, indicating a significantly elevated risk. However, when comparing seropositive and seronegative RA patients, the difference in lung cancer risk was not substantial. A stratified analysis revealed that the increased risk was particularly pronounced among the male patients (interaction *p* < 0.001) and those with a history of heavy smoking (interaction *p* = 0.046), both conditions being satisfied by our patient. In contrast, age and the presence of interstitial lung disease did not significantly influence the association between RA and lung cancer risk [18].

In contrast, Wang, L. and colleagues recently published a study indicating that there is no causal association between RA and NSCLC. However, the therapeutic agents used in the treatment of RA, particularly tumor necrosis factor (TNF) inhibitors, may potentially increase the risk of developing NSCLC [19].

Our patient’s CT findings, with right, latero-thoracic thickening of the pleura, were highly suggestive of pleural mesothelioma, which was initially raised as the primary diagnostic suspicion. Differentiating pleural metastases from malignant pleural mesothelioma (MPM) remains a frequent diagnostic challenge in thoracic imaging. On the chest CT, metastatic pleural disease characteristically presents with moderate- to large-volume, free-flowing effusions, often accompanied by focal or diffuse pleural nodularity and the associated pulmonary metastases or lymphadenopathy. In contrast, MPM is suggested by the circumferential pleural thickening, pleural masses, and direct invasion of adjacent structures. Additionally, the CT findings of asbestos-related pleural disease, including pleural plaques, were exclusively identified in the MPM and not in metastases [20]. Recent comparative CT analyses further support these distinctions and even inform machine learning-based models to aid in differentiating MPM from metastatic pleural disease [21]. Therefore, while pleural metastases should remain the leading diagnosis in most cases, specific imaging markers—especially circumferential thickening, pleural masses, asbestos-related findings, and organ invasion—are essential to raise suspicion for MPM and prompt appropriate histopathological confirmation. The thickening of the right pleura was caused by metastatic lesions, contrary to our initial suspicion of pleural mesothelioma.

Another potential diagnosis to be considered is that of primary pleural adenocarcinoma. However, Shalahuddin et al., in their study of pleural metastasis, were unable to find cases of primary pleural adenocarcinoma reported in the literature, with the closest being that of primary pleural squamous cell carcinoma [22]. Even though both metastatic lesions and mesothelioma can cause pleural thickening, Kim et al. discovered that circumferential thickening, defined as continuous thickening involving greater than three-quarters of the pleura, is more related to mesothelioma [23]. In this way, our case report sets to present the multiple facets of lung adenocarcinoma, its complex differential diagnosis, and the radiologic challenges in assessing the etiology of pleural lesions.

The diagnosis could only be assessed by histopathological examination, thus, with the help of our thoracic surgery department, a right pleural biopsy was performed, which revealed carcinomatous infiltrates with a solid and tubular architecture, composed of atypical epithelial cylindrical cells with mucin secretion, suggestive of adenocarcinoma. In addition, the bronchitic biopsy was also suggestive of adenocarcinoma, with the diagnosis being further confirmed by an immunohistochemical analysis. The primary site of the tumor appears to be pulmonary in origin.

Despite a better outcome compared to pleural mesothelioma, our patient’s prognosis remains reserved, considering the advanced stage of the disease and the unfavorable medical history.

Due to the patient’s autoimmune background, the differential diagnosis in our case had to take into account the possibility of rheumatoid pulmonary nodules. Evidence suggests that rheumatoid pulmonary nodules are more commonly found in younger individuals with a lower cumulative smoking history than those diagnosed with malignant nodules [24]. In some cases, the nodules can be compared to malignant lesions on imaging, particularly when they are subpleural, cavitated, or enlarging rapidly. Even so, smooth margins, the absence of significant growth, and relative clinical stability frequently indicate a benign cause [24]. In comparison, malignant tumors tend to develop irregularly, with a gradual progression, and may be linked to other symptoms like lymphadenopathy or pleural obstruction [24]. Imaging findings may not always be conclusive, especially in complex cases where multiple comorbidities and immunosuppressive therapies may mask the typical patterns. To prevent misclassification, a tissue biopsy is still required for an accurate diagnosis in such cases.

The absence of histopathological confirmation of the iliac lesion represents a diagnostic limitation. While its radiologic features strongly suggest a metastatic etiology, definitive classification remains pending. Plans for tissue confirmation, including an image-guided biopsy or follow-up imaging, are under consideration as part of the ongoing diagnostic strategy, depending on the patient’s clinical evolution and performance status. Acknowledging this uncertainty is crucial for ensuring accurate staging and guiding appropriate management.

Chronic inflammatory diseases such as RA, psoriatic arthritis, and systemic lupus erythematosus are frequently treated with DMARDs. Although they can control inflammation and slow disease progression, the long-term effects of these agents on malignancy are still a concern due to their immunosuppressive and immunomodulatory properties. The primary therapeutic effect of DMARD, which is a synthetic compound, is demonstrated by the inhibition of dihydrofolate reductase and changes in immune function through enhanced cellular ATP signaling and suppressed T- and B-cell activity. Despite its association with an elevated risk of non-melanoma skin cancers and lymphoproliferative disorders, particularly EBV-related lymphocytes, methotrexate may have a neutral or protective effect on specific solid tumors such as colorectal and hepatocellular carcinomas [25].

## 4. Conclusions

This case illustrates the complex interaction between chronic systemic inflammation, long-term immunosuppressive therapy, and increased oncologic risk, particularly in the context of lung cancer.

Chronic hypoxemia secondary to COPD favored the development of a prothrombotic milieu, further enhanced by the underlying neoplastic disease. The cumulative presence of additional well-established risk factors for a venous thromboembolism including advanced age (>60 years), prolonged immobilization due to joint pain associated with rheumatoid arthritis, and osteomuscular metastasis involving the right hip contributed to the occurrence of a bilateral pulmonary embolism. Whether arising as a complication in the setting of multimorbidity or as a potential paraneoplastic phenomenon, the pulmonary embolism was the key event responsible for the acute clinical decompensation and onset of respiratory failure that led to the hospital admission.

Studies have shown that the incidence of lung cancer is significantly higher in patients with RA, but despite this association, no direct causal relationship between RA and NSCLC has been identified. As for the therapeutic agents used in the treatment of rheumatoid arthritis, such as TNF inhibitors, they may contribute to an increased risk of developing non-small cell lung cancer. Additionally, conventional DMARDs have been linked to a higher incidence of both malignancy and interstitial lung disease. These findings highlight the need for careful risk–benefit assessments and long-term monitoring in patients undergoing immunosuppressive therapy.

## Figures and Tables

**Table 1:** Arterial blood gas analysis.

	T1	T2
pH	7.476	7.497
PaO_2_	54.8 mmHg	58 mmHg
PaCO_2_	32.5 mmHg	39.5 mmHg
SaO_2_	87% (room air)	92.1% (room air)
HCO_3_	25.1 mmol/L	30.3 mmol/L
Lactate	1.4 mmol/L	1.4 mmol/L
Glucose	110 mg/dL	117 mg/dL

T1—at admission. T2—at discharge.

**Table 2:** Laboratory blood tests.

Laboratory Tests	Values	Normal Range
WBC (×10^3^/µL)	16.55	3.9–10.9
NEUT (×10^3^/µL)	13.70	1.8–6.98
LDH (U/L)	809	140–280
CRP (mg/L)	138	0–5
ESR (mm/h)	33	2–20
Fibrinogen (mg/dL)	555	200–400
Serum Iron (ng/dL)	32.8	50–150
Direct Bilirubin (mg/dL)	0.47	0.1–0.3
Rheumatoid Factor (U/mL)	22.1	<20

**Table 3:** Respiratory functional tests.

Values	T1	T2
FVC%	43.5	50.3
FEV1%	28.7	34.6
TI%	51.67	53.99
MEF50%	10.5	14
Interpretation	Very severe mixed respiratory dysfunction	

T1—Initial spirometry. T2—Reassessment after a few days.

## Data Availability

The original contributions presented in this study are included in the article. Further inquiries can be directed to the corresponding author.
